# Rare Coexistence of a Single Coronary Artery, Myocardial Bridging, and Bicuspid Aortic Valve Detected by Coronary Computed Tomography Angiography During Preoperative Assessment: A Case Report and Literature Review

**DOI:** 10.3390/reports9020156

**Published:** 2026-05-19

**Authors:** Piotr Machowiec, Piotr Przybylski, Elżbieta Czekajska-Chehab

**Affiliations:** Department of Radiology, Medical University of Lublin, 20-059 Lublin, Poland; dr.przybylski@gmail.com (P.P.); czekajska@gazeta.pl (E.C.-C.)

**Keywords:** single coronary artery, bicuspid aortic valve, myocardial bridging, cardiac computed tomography

## Abstract

**Background and Clinical Significance**: Bicuspid aortic valve (BAV) is the most common congenital heart defect and may coexist with other cardiovascular anomalies. Among these is a single coronary artery (SCA), a rare congenital condition in which the entire coronary circulation originates from a single coronary ostium. Cardiac computed tomography (CCT) enables simultaneous evaluation of coronary artery anatomy and aortic valve morphology with high spatial resolution, which may influence procedural strategy in patients undergoing valve interventions. **Case Presentation**: This report represents the first documented case of a 59-year-old male with mixed aortic valve disease in whom preoperative CCT revealed the coexistence of BAV, SCA (Lipton type L-I), and myocardial bridging (MB) involving the mid segment of the left anterior descending artery (LAD). Identification of these findings was crucial for preoperative assessment and contributed to the selection of an appropriate surgical strategy. **Conclusions**: CCT plays a key role in the preoperative evaluation of valvular heart disease, including in patients with coexisting BAV and SCA. It enables individualized procedural planning and minimizes the risk of perioperative complications.

## 1. Introduction and Clinical Significance

The coexistence of the majority of coronary artery anomalies with bicuspid aortic valve (BAV) has been well described in the literature [1]. In contrast, limited data are available regarding the association of BAV with a single coronary artery (SCA) as well as myocardial bridging (MB) of epicardial arteries. SCA is a congenital anomaly characterized by the presence of a single coronary artery originating from a solitary coronary ostium, with a reported prevalence ranging from 0.024% to 0.066% [2]. Most cases of SCA are asymptomatic. However, a malignant variant occurs when the coronary artery courses between the aorta and the pulmonary artery, a configuration associated with an increased risk of sudden cardiac death and other complications [3,4]. According to the Lipton classification, isolated SCA variants are divided into three main groups (I–III), further designated by the letter R or L depending on the origin of the single coronary artery [5]. Patients with BAV and concomitant mixed aortic valve disease, including both aortic stenosis and aortic regurgitation, as well as a congenital SCA, may present with symptoms of myocardial ischemia and heart failure [6]. Furthermore, MB is a congenital condition in which a segment of a coronary artery courses intramyocardially, with a reported prevalence ranging from 3.5% to 58%. Although typically an incidental finding with a favorable prognosis, specific anatomical and hemodynamic features of MB may be independently associated with an increased risk of myocardial ischemia, arrhythmias, and other adverse cardiovascular events [7].

Cardiac-CT with ECG gating (CCT-ECG) enables simultaneous evaluation of the coronary arteries and aortic valve morphology, providing higher diagnostic accuracy and superior spatial resolution compared with echocardiography [8]. It is also a noninvasive method for assessing the location, depth, and length of myocardial bridging [7]. We present the case of a patient with mixed aortic valve disease and a rare constellation of cardiovascular anomalies incidentally detected on CCT-ECG, including SCA with associated MB and BAV. This case highlights the value of CCT-ECG in the preoperative assessment of patients undergoing evaluation for aortic valve intervention and, to the best of our knowledge, constitutes the only such case reported in the available literature.

## 2. Case Presentation

**Figure 1:**
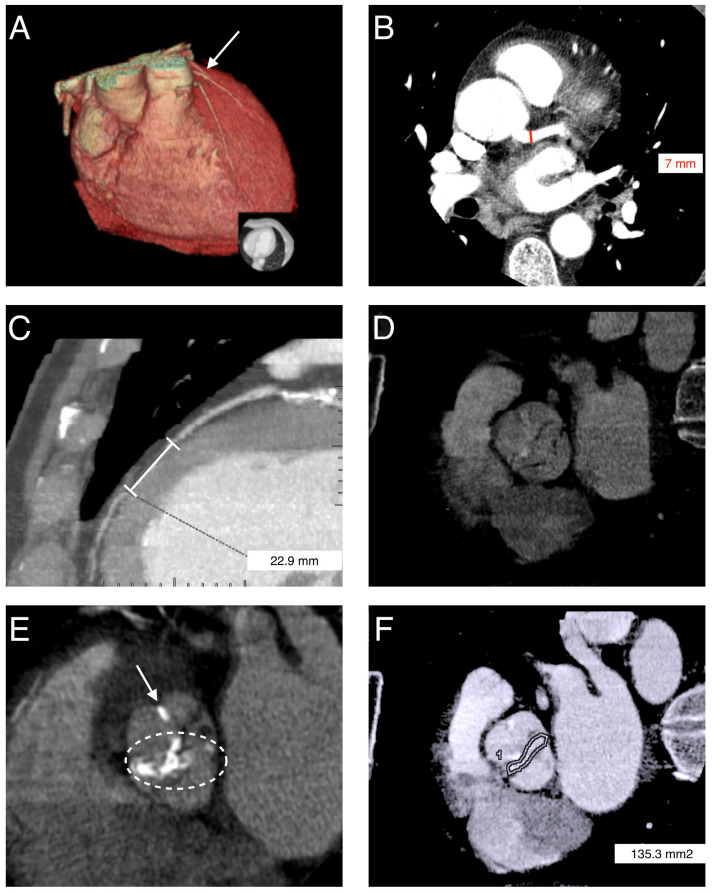
(**A**) Three-dimensional volume-rendered (3D-VR) image illustrating SCA (white arrow). (**B**) Axial image demonstrating a relatively wide LMCA, measuring 7 mm in diameter (red line). (**C**) Dimension line indicating MB in the left anterior descending artery (LAD), approximately 23 mm in length, with mild luminal narrowing. (**D**) BAV during opening—type 1 R–L. (**E**) BAV with extensive calcifications (within the white loop) and a calcified raphe between the coronary cusps (type 1 R–L)—white arrow. (**F**) Aortic valve opening area measured at 25% of the R–R interval—135.3 mm^2^.

A 59-year-old man with a clinically known mixed aortic valve disease, previously diagnosed on echocardiography and consisting of severe aortic stenosis with grade IV aortic regurgitation, was referred for CCT-ECG prior to planned aortic valve replacement. The examination was performed due to the inability to catheterize the right coronary artery (RCA) during invasive coronary angiography and the atypical perfusion territory of the left coronary artery. The patient did not report typical anginal symptoms. Transthoracic echocardiography also demonstrated reduced left ventricular ejection fraction (approximately 45%) and left ventricular enlargement. CCT was performed using a retrospective ECG-gated acquisition protocol, including native scanning followed by intravenous administration of contrast medium. The examination demonstrated the absence of a coronary ostium in the right sinus of Valsalva. The left main coronary artery (LMCA) had a typical origin and branching pattern and measured approximately 7 mm in diameter. The left circumflex artery (LCx) demonstrated an atypical course; it ran within the atrioventricular groove along the inferoposterior surface of the heart and subsequently following a trajectory corresponding to the mid and distal segments of RCA. Based on CCT-ECG findings, a coronary anomaly in the form of SCA was diagnosed, classified as type L-I according to the Lipton classification. A malignant variant of SCA was excluded. No significant coronary artery stenoses were identified. In addition, MB involving the mid-segment of the left anterior descending artery (LAD) was detected (segment 7 according to the Society of Cardiovascular CT) [9], extending over a length of 23 mm and causing mild luminal narrowing (Figure 1).

An incidental finding on CCT-ECG was the presence of BAV with fusion of the right and left cusps, corresponding to type 1 R–L according to the Sievers–Schmidtke classification [10]. Moderate leaflet thickening and extensive calcifications of the aortic valve were observed, with imaging features consistent with aortic stenosis and aortic regurgitation. The aortic valve area was 135 mm^2^ (Figure 1). Additionally, CCT-ECG confirmed the substantial left ventricular enlargement previously observed on echocardiography, with an end-diastolic volume (EDV) of 327 mL and an ejection fraction (EF) of 43%. The patient subsequently underwent surgical aortic valve replacement (SAVR) with implantation of a mechanical prosthesis, with a favorable clinical outcome. Following surgery, the patient remains under regular follow-up in both cardiology and cardiac surgery outpatient clinics, including periodic clinical and echocardiographic evaluation as well as monitoring of coagulation parameters during chronic therapy with a vitamin K antagonist.

## 3. Discussion

The coexistence of BAV and SCA in the setting of mixed aortic valve disease is extremely rare, although its exact epidemiology remains unknown. To date, no standardized management algorithms have been established for patients with surgically indicated aortic valve disease and coexisting SCA. The available evidence is derived primarily from isolated case reports [4,11,12,13]. Selection of the optimal therapeutic approach should take into account several factors, including the type and severity of the valvular lesion, the diameter of the ascending aorta, the anatomical origin and course of the SCA, and the presence of features suggestive of myocardial ischemia.

In the present patient, taking into account the anatomical considerations, the presence of relatively extensive calcifications along the edges of the BAV cusps, as well as the institutional experience and standard clinical practice, surgical aortic valve replacement with implantation of a mechanical prosthesis was selected. After completion of the necessary imaging studies, including preoperative CCT-ECG, no dilation of the ascending aorta, malignant course of the SCA, or other coronary anomalies requiring modification of the planned valve procedure were identified. Furthermore, MB observed in the mid-segment of the LAD appeared hemodynamically and clinically insignificant. MB is generally considered a benign entity that does not impair coronary blood flow and does not require therapeutic intervention [7].

In a study by Giebels et al., the importance of detailed preoperative imaging prior to heart valve surgery was emphasized. In a patient undergoing BAV replacement, preoperative computed tomography revealed a single coronary ostium located in the right sinus of Valsalva. The LMCA followed a long course along the right margin of the aorta (consistent with a Lipton type R-III single coronary artery). To facilitate the planned annuloplasty, the LMCA was reimplanted into the noncoronary sinus. Giebels et al. highlighted that preoperative CT allows optimal planning of annular dimension normalization and commissural orientation, which are key elements for successful BAV repair [11].

Mohammad et al. noted that the coexistence of SCA and BAV is not always associated with the presence of aortic stenosis and/or regurgitation requiring surgical intervention. In the case presented by the authors, CCT performed during routine evaluation of chest pain incidentally revealed an aneurysm of the sinus of Valsalva, which was successfully repaired using a synthetic patch [4]. Additionally, the patient presented with a Lipton type R-III single coronary artery, which is one of the least common SCA variants. This case suggests that the coexistence of BAV and SCA may be asymptomatic or only mildly symptomatic without impairment of aortic valve function. However, in some individuals it may be associated with the presence of additional cardiovascular anomalies.

To date, only a few cases of transcatheter aortic valve implantation (TAVI) have been reported in patients with SCA and severe aortic stenosis. Patients undergoing TAVI are exposed to a risk of coronary artery occlusion (approximately 1%), which may be greater in the presence of a single coronary ostium. Dursun et al. emphasized that a self-expanding valve may represent the preferred option in patients with SCA undergoing TAVI, particularly because of its recapturability [12]. Furthermore, Bertin et al. reported the first case of TAVI performed in a patient with both SCA (consistent with a Lipton type R-II single coronary artery) and BAV. The procedure was successful, and the patient was discharged without complications [13]. The authors emphasized that, in addition to the risks associated with SCA, TAVI in patients with BAV poses a technical challenge related to valve positioning and expansion due to the asymmetric configuration of the aortic annulus and extensive leaflet calcification. Moreover, bicuspid aortic valve should be regarded as a heterogeneous spectrum of anatomical phenotypes, including variable cusp fusion patterns and associated aortopathy, which may further influence procedural planning, prosthesis sealing, and long-term durability following TAVI [14]. The presented patient had type 1 R–L BAV according to the Sievers–Schmidtke classification, with severe calcifications involving the valve cusps. Yoon et al. demonstrated that in patients with bicuspid aortic valve treated with newer-generation TAVI devices, calcified raphe and excessive leaflet calcification were associated with an increased risk of procedural complications and adverse mid-term outcomes. Patients presenting these anatomical features experienced higher rates of aortic root injury, moderate-to-severe paravalvular regurgitation, and increased 30-day mortality compared with those without such morphological characteristics [15]. Barbanti et al. highlighted that crossing a bicuspid aortic valve can be technically more difficult due to its anatomy, such as the presence of a raphe in type 1 and 2 BAV or two large cusps in type 0 BAV. This often necessitates the use of additional wires, catheters, and procedural maneuvers [14]. On the other hand, in a study by Li et al., patients with type 0 BAV were shown to have a more favorable long-term prognosis compared with those with TAV and type 1 BAV. This finding suggests that the absence of a raphe and a more symmetrical valve morphology may enable more uniform prosthesis expansion, in contrast to the asymmetrical displacement typically observed in raphe-dominant anatomies [16].

In addition, anatomical variability also affects device selection and sizing strategies. Different approaches have been proposed to account for these challenges, including dedicated supra-annular and annular sizing methods, which integrate CT-derived measurements to better predict prosthesis behavior within the native valve anatomy [17,18,19]. Taken together, these considerations highlight the importance of meticulous multimodality imaging and individualized procedural planning to optimize outcomes in this complex patient population. Several features have been reported as high-risk or technically challenging for TAVI procedures, including heavily and asymmetrically calcified raphes, excessive leaflet or annular calcification, extremely large annular dimensions, shallow or effaced sinuses of Valsalva, extensive LVOT calcification, and marked aortopathy, in which cases surgical aortic valve replacement may be the preferred treatment strategy [20]. In the patient presented in our report, SAVR was performed primarily because of the presence of heavily calcified raphes and severe leaflet calcifications. In the remaining patients with type 1 BAV who do not meet the aforementioned exclusion criteria, TAVI may still represent a feasible treatment option. In such cases, supra-annular measurements should be considered for valve sizing, while the use of self-expanding valves may be preferable to achieve a larger effective orifice area (EOA) [20].

Computed tomography plays a pivotal role in guiding transcatheter heart valve (THV) sizing in patients with BAV while also providing detailed anatomical assessment that facilitates appropriate valve selection. Balloon-expandable valves may provide more optimal expansion and sealing properties, whereas self-expanding valves are associated with a lower risk of annular injury and may result in lower transvalvular gradients due to their supra-annular design [14]. Furthermore, intra-annular balloon-expandable valves may represent an optimal option for patients with more symmetric anatomies; however, meticulous sizing is required to avoid oversizing and its potential complications [20].

Maintaining durable coronary access and ensuring the feasibility of selective cannulation post-transcatheter aortic valve implantation is of paramount clinical importance, particularly in patients with rare anatomical variations like a single coronary artery [21]. Procedural strategies, specifically commissural alignment, seem to be critical in SCA patients to avoid positioning a stent post directly in front of the single ostium [22]. According to Emmanuel et al., valve selection is primarily guided by anatomical suitability and procedural safety. Balloon-expandable valves are favored in patients with suitable coronary anatomy, particularly adequate coronary height and anticipated easy coronary re-access in the event of complications. In contrast, some operators prefer self-expanding valves because their recapturable design allows repositioning until approximately two-thirds of the prosthesis is deployed, potentially providing an additional safety advantage during intraprocedural complications [13,23]. To reduce the risk of coronary obstruction in patients with SCA, some operators perform angiography during balloon valvuloplasty to assess potential coronary compromise, while others place a protective coronary wire in the single coronary artery during balloon valvuloplasty and transcatheter valve implantation [24,25]. In the general population, it has been shown that the use of supra-annular, tall-frame valves, particularly when combined with a high TAV-to–Sinus of Valsalva relation and a deep implantation depth, may result in unsuccessful coronary cannulation, as the coronary ostium can be directed toward the lower, sealed portion of the stent frame. This configuration is particularly unfavorable in patients with SCA, in whom maintaining reliable coronary access is of critical clinical importance [26]. On the other hand, self-expandable valves might better adapt to the irregularly shaped landing zone of BAV [27]. In such cases, the use of transcatheter heart valves with a low-frame profile, or alternatively the application of commissural alignment techniques in supra-annular devices, may improve the likelihood of successful selective coronary engagement [28]. However, there is no single standardized algorithm for the management of patients undergoing TAVI with concomitant BAV and SCA. Management in such scenarios should therefore be individualized, taking into account detailed anatomical characteristics and procedural risk factors.

Accurate preoperative identification of the aortic valve morphology is essential for determining the optimal therapeutic strategy in aortic stenosis. CCT-ECG enables simultaneous evaluation of the coronary arteries and aortic valve morphology, providing substantially greater diagnostic accuracy compared with other imaging modalities [8]. This is particularly important in patients with BAV, as it often necessitates modifications to the surgical approach and consideration of potential concomitant cardiovascular defects, including coronary anomalies. This case demonstrates that BAV and associated coronary anomalies may be incidentally detected during preoperative evaluation using CCT-ECG, highlighting its clinical utility and diagnostic value.

## 4. Conclusions

To the best of our knowledge, this is one of the first reported cases of concomitant mixed aortic valve disease in a patient with BAV, SCA, and MB involving one of its branches, identified on CCT-ECG.

CCT-ECG plays a key role in the preoperative evaluation of valvular heart disease, including in patients with coexisting BAV and SCA. It enables individualized procedural planning and minimizes the risk of perioperative complications.

Patients with SCA may remain asymptomatic for many years, and the anomaly is often detected incidentally during imaging studies. However, in some individuals—particularly when the artery follows an interarterial course between the aorta and the pulmonary trunk—the anomaly may present with angina, syncope, arrhythmias, and, in rare cases, sudden cardiac death.

## Data Availability

The original data presented in this study are available upon reasonable request from the corresponding author. The data are not publicly available due to privacy concerns.
